# The use of an external fixator system with Hey Groves bone clamps in transverse femoral shaft fractures

**DOI:** 10.1308/003588412X13373405386015d

**Published:** 2012-09

**Authors:** MC Quaye, CM Jordan, AJ Jowett

**Affiliations:** Portsmouth Hospitals NHS Trust,UK

## BACKGROUND

Intramedullary fixation for subtrochanteric femoral fractures has gained popularity in recent years. Obtaining and holding adequate reduction in order to pass the guidewire can pose a major obstacle when there are significant deforming forces on the proximal fragment.^1^ This can necessitate a mini open procedure to align the distal and proximal fragments. In short oblique or spiral fractures, careful application of Hey Groves bone holding forceps encircling both fragments can hold the reduction successfully. However, in transverse fractures, a single clamp will not suffice. Assistance is often required to align the proximal and distal segments with two forceps in order to pass the guidewire. We describe a technique allowing a single surgeon to hold adequate alignment without requiring assistance.

## TECHNIQUE

**Figure 1:**
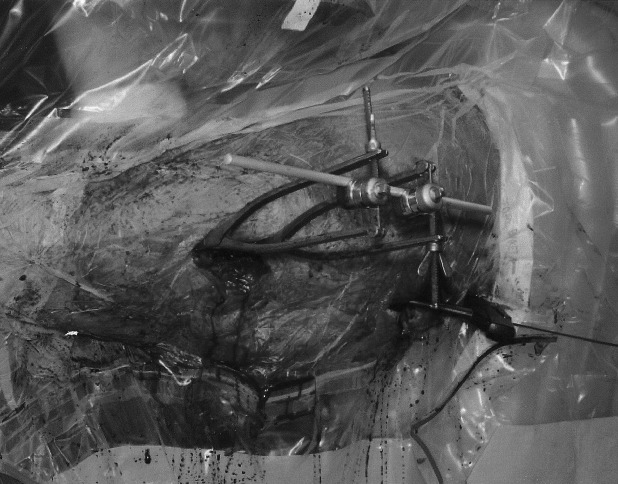
Intra-operative use of fixator

**Figure 2:**
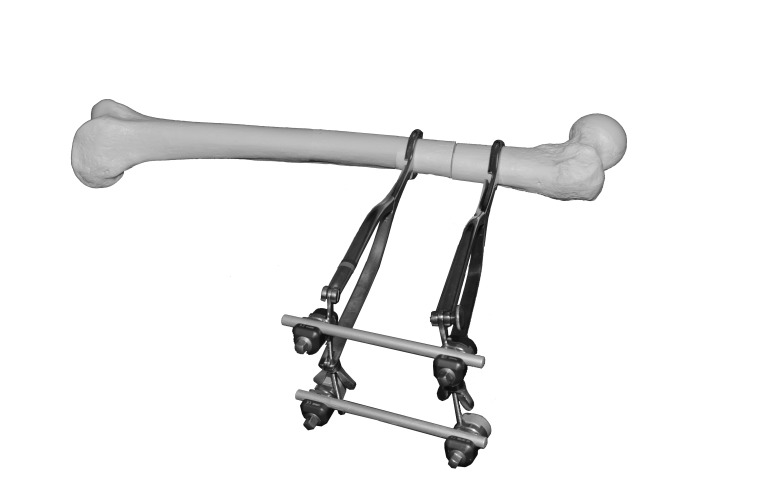
Dry bone model of device application

A small incision is made to allow the applications of two Hey Groves clamps to the distal and proximal segments. Two rod-to-rod external fixator couplings are attached to the ratchet of the forceps and a rod is used the span the two (Fig 1). Once adequate reduction is obtained, the clamps are tightened to hold the position. If a large deforming force is present, one can add two further clamps and a bar to stiffen the construct (Fig 2).

## DISCUSSION

This simple technique can assist the single surgeon with difficult reductions, allowing easy passage of the femoral intramedullary guidewire. We used the Hoffman II® external fixation system (Stryker, Kalamazoo, MI, US) although most commonly used trauma external fixation systems can be employed.
